# Improved Generation, Physicochemical Characteristics, and Food Application Studies of a Red Colorant Obtained from Oxidative Coupling of Chlorogenic Acid and Tryptophan

**DOI:** 10.3390/foods13050686

**Published:** 2024-02-23

**Authors:** Ardemia Santarcangelo, Nadine Schulze-Kaysers, Andreas Schieber

**Affiliations:** Institute of Nutritional and Food Sciences, Molecular Food Technology, Agricultural Faculty, University of Bonn, 53115 Bonn, Germany; santarcangelo@uni-bonn.de (A.S.); nadine.schulze@uni-bonn.de (N.S.-K.)

**Keywords:** chlorogenic acid, red pigment, optimized synthesis, stability, design of experiment

## Abstract

Due to a widespread consumer reluctance toward synthetic food dyes, the interest in natural compounds from plants has increased. This study aimed to optimize the oxidation process between chlorogenic acid (CQA) and tryptophan (Trp) using sodium periodate (NaIO_4_) to obtain a red-colored pigment. The impact of temperature and different ratios of Trp to NaIO_4_ on the reaction progress was investigated. After the best conditions for the reaction were established, three pH values were tested. The reaction time could be reduced from 72 to 24 h with a yield of 46 ± 2% *w*/*w* based on the quantity of CQA. After the first purification step of the product by size exclusion chromatography, the pigment obtained was characterized for its solubility, and its hydrolyzed form was used for investigations into the stability at different pH values, storage under light and in the dark (period of 28 days), in the presence of reducing agents, and for heat resistance. Finally, several food matrices were successfully colored with the natural pigment in amounts from 0.005 to 0.01% (*w*/*w*). In conclusion, the present study provides new insights into the feasible production and comprehensive characterization of a red pigment derived from oxidative coupling of CQA and Trp, as well as its application in food systems.

## 1. Introduction

There is a marked reservation among consumers about synthetic additives, including artificial colors [1,2,3]. Therefore, great efforts are currently being made to find natural colorants for use in foods. Since colorants of animal origin are not acceptable to vegetarians and vegans, substances from plant sources, in particular, such as anthocyanins, betalains, and carotenoids, are at the forefront of interest [4]. However, these classes of pigments are characterized by a more or less high sensitivity to external influences such as thermal treatment, exposure to oxygen and light, and changes in pH values [5,6,7,8,9]. 

In view of the aforementioned challenges, it is noteworthy that the oxidative coupling of natural substances, namely chlorogenic acid and amino acids, results in the formation of colored reaction products [10,11,12]. While in these reactions, the majority of amino acids form green pigments, representing substituted benzacridines, a red colorant is obtained with tryptophan [13,14]. 

**Figure 1:**
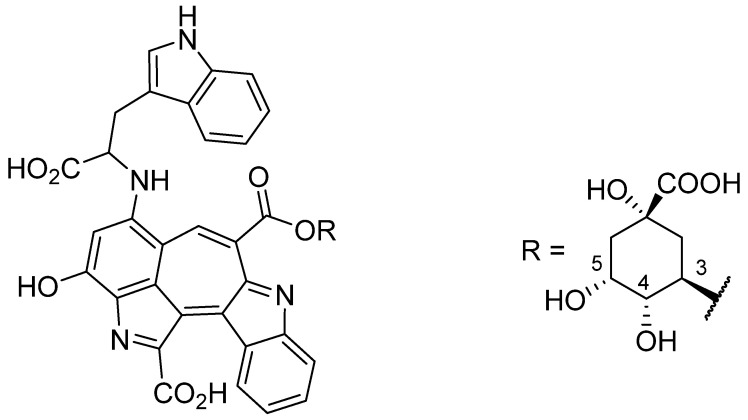
Structure of a red compound consisting of a chlorogenic acid moiety, in which the caffeic group is esterified with a hydroxyl group of quinic acid, attached to two tryptophan moieties, one of which is embedded in the chromophore core. While the caffeic acid moiety can be attached to positions 1, 3, 4, and 5, only one isomer is shown in this representation.

After Moccia et al. [15] had described two red compounds based on cyanines, our own studies demonstrated the formation of different red products consisting of one equivalent of chlorogenic acid with the quinic acid still attached and two equivalents of tryptophan [16]. The structure of the predominant compound is shown in Figure 1. Because the focus of that study was on the structural elucidation of the pigment and the proposal of a formation pathway, no investigations into the physicochemical properties and stability were performed. Furthermore, the formation of the pigment required a reaction time of 72 h, which is not acceptable in terms of economic production. Therefore, the aim of the present study was to develop a process for the accelerated generation of the red colorant and to characterize its physicochemical properties, such as solubility in various solvents and stability against heat and light. In addition, the colorant was used in select food systems to evaluate its application potential.

## 2. Materials and Methods

### 2.1. Chemicals

Chlorogenic acid (CQA; ≥95%) was obtained from BLDpharm (Shanghai, China). L-Tryptophan (≥98%), sodium metaperiodate (NaIO_4_), sodium hydroxide, L-cystein (≥97%), and ascorbic acid (≥99%) were purchased from Sigma-Aldrich (St. Louis, MO, USA). Carmine (E 120, ≥43%) was acquired from Symrise AG (Holzminden, Germany). Hydrochloric acid was purchased from VWR International GmbH (Darmstadt, Germany).

### 2.2. Optimization of Pigment Production by Means of Experimental Design

The oxidation of CQA was performed according to protocols reported previously [16], in which a solution of Trp (112 mM) with NaIO_4_ (7 mM) and CQA (28 mM) buffered in Tris buffer (0.2 M; pH 7.5) was mixed (1:1, *v*/*v*), obtaining final concentrations of 56 mM Trp, 3.5 mM NaIO_4_ and 14 mM CQA.

The process optimization for an increase in red pigment yields was conducted using the software Design Expert version 9.0.6.2 (Stat-Ease, Inc., Minneapolis, MN, USA). First, a full factorial design with 3 center points (Table S1) was employed to screen the effects of three independent variables (concentrations of Trp and NaIO_4_, temperature) on the dependent variables (peak area at 550 nm after 24, 48, and 72 h). A temperature range of 22–70 °C and a range of 24–72 h for the reaction time was chosen. Trp and NaIO_4_ were used in ranges of 56–140 mM and 1.68–14 mM, respectively (referred to as the final solution). Based on the findings of this screening, an increase in temperature was no longer considered since higher temperatures led to a decrease in the peak area of the three compounds detected by UHPLC at 550 nm. In the next step, the effect of the concentrations of Trp and NaIO_4_ (56–140 mM and 3.50–14 mM, respectively) was analyzed in a factorial design with 4 center points (Table S2), resulting in 12 experiments.

Based on the results of an initial set of experiments, NaIO_4_ at a starting concentration of 3.5 mM was used because it was found that larger quantities correlate with a larger peak area for the three compounds at 550 nm. This concentration also corresponds to that used in our previous study [16] and thus served as a reference point for the optimization process. Due to the significance of the curvature in the adjusted model, this design was augmented with face-centered axial points for optimization (Table S3). In all screening and optimization experiments, the reaction was carried out under vigorous stirring (40 rpm) using a Variomag Thermomodul 40 ST (HP Labortechnik, Oberschleißheim, Germany) for 72 h in a volume of 10 mL. Samples were taken after 24, 48, and 72 h, diluted with water (1:10; *v*/*v*), and analyzed by UHPLC. 

Because the optimization showed an increase in the amount of red pigment with increasing NaIO_4_ concentrations, additional experiments were conducted with 21, 28, and 42 mM NaIO_4_. Based on the optimal parameter settings, the reaction was subsequently performed in 0.1 M acetate buffer solution at pH 5 or in 0.2 M Tris buffer solution at pH 7.5 and 9. The resulting red pigment was precipitated by acidification to pH 1 with 6 M HCl and freeze-dried after centrifugation (10,000 rpm, 15 min, 4 °C). As a control, the reaction was carried out under optimal conditions but without NaIO_4_. All reactions were performed in duplicate.

### 2.3. Scale up and Isolation of the Red Pigment

In another set of experiments, the reactions were performed as described in Section 2.2. under the optimized conditions (14 mM CQA, 56 mM Trp, 14 mM NaIO_4_, 22 °C, pH 9 in 0.2 M tris buffer) but on a larger scale. For this purpose, CQA (0.25 g) was reacted with Trp (0.57 g) in the presence of NaIO_4_ (0.15 g) in a final volume of 50 mL Tris buffer (pH 9, 0.2 M). After acidification to pH 1 with 6 M HCl, a precipitate was formed, which was separated by centrifugation (10,000 rpm, 15 min, 4 °C) and subsequently lyophilized. The precipitate was dissolved in 0.2 M NaOH and then purified on Sephadex G-10 (46 × 3.5 cm) using water as an eluent. Fractions of 10 mL were collected, diluted with water (1:50, *v*/*v*), and analyzed spectrophotometrically. The fractions showing an absorption maximum at approximately 550 nm were combined, and the solution was acidified to pH 1 using 6 M HCl. After centrifugation, the precipitate was collected and freeze-dried. In total, 0.114 ± 0.004 g of a red powder was obtained, corresponding to a yield of 46 ± 2% (*w*/*w*) based on CQA. The above-described protocol was highly reproducible and was therefore used when additional material was required for application studies.

### 2.4. Solubility Tests

The solubility of the colorant in water, alkaline solution (0.06 M NaOH), and ethanol was determined using 5 mg of the pigment. It turned out that it could be solubilized only in sodium hydroxide and ethanol, whereas its water solubility was poor. For subsequent analysis by HPLC, the pigment was dissolved first in NaOH or in ethanol and afterwards diluted (1:100; *v*/*v*) in water or ethanol. 

### 2.5. Stock Solution of the Hydrolyzed Pigment

A stock solution of the hydrolyzed pigment, that is, the core molecule without the quinic acid moieties initially attached to chlorogenic acid, was obtained by dissolving 5 mg of pigment in 0.06 M NaOH (1.7 mL) for 4 h. This stock solution was used for subsequent investigations.

### 2.6. Stability at Different pH Values

The stock solution prepared in Section 2.5 was diluted (1:100; *v*/*v*) in 0.1 M phosphate buffer at pH 7 and 8 and in 0.1 M acetate buffer (pH 3 and 5). The absorbance of the solutions was determined by U*V*/*V*is spectrophotometry in the range from 400 to 800 nm.

### 2.7. Storage Stability

The samples prepared as described in Section 2.6 were capped, sealed with parafilm, and divided into two groups. One sample was exposed to direct daylight, whereas the other was stored in darkness. Both groups were kept at room temperature, and the absorbance was measured after 1, 7, 14, and 28 days in the range from 400 to 800 nm.

### 2.8. Thermal Stability

For the determination of the thermal stability, the stock solution prepared as described in Section 2.5 was diluted 1:100 (*v*/*v*) in 0.1 M phosphate buffer at pH 7 and in 0.1 M acetate buffer at pH 3.6 and 5 (0.029 mg/mL) and placed in capped vials. These were sealed with parafilm, kept at 90 °C for 30 min, and periodically analyzed by UV-Vis spectroscopy at 10-min intervals. For comparison, the thermal stability of a commercial carmine colorant (0.059 mg/mL) was determined using the same procedure.

### 2.9. Stability in the Presence of Reducing Agents

Aliquots of 10 μL of the stock solution (Section 2.5) were added to 880 μL of 0.1 M phosphate buffer (pH 7), to 0.1 M acetate buffer (pH 3.6), or to 880 μL water, to which 100 μL of a solution of ascorbic acid or cysteine (10 mg/mL) was added subsequently. A solution prepared with 100 μL water instead of the reducing agents served as a control. The samples were kept in the dark at room temperature, and their absorbance was determined by comparing the absorbance values obtained from the UV-Vis spectrum after 0, 2, 5, and 24 h.

### 2.10. Application as a Food Colorant

The pigment was used as a coloring agent in several model food applications. For this purpose, 40 mg of the colorant was dissolved in 12.8 mL NaOH (0.06 M), agitated for 4 h, and subsequently stored in a brown glass bottle at room temperature. Aliquots of this stock solution were added to the model foods as described below.

Milk and plant-based alternatives: 500 μL of the stock solution was mixed with 15 mL of cow´s milk or oat drink and compared with the respective controls without added colorant. 

Yogurt: 500 μL of the solution was blended with 30 g of natural yogurt (3.5% fat) and compared with the respective control without adding colorant. 

Meringue: 1500 μL of the solution and a pinch of salt were added to 60 g of egg white and whisked. Subsequently, 110 g of sucrose was gradually added until stiff peaks occurred. The meringues were shaped with a piping bag and dried at 90 °C in an oven for 2 h.

The colored samples were stored in a refrigerator (4 °C). Color analysis on all samples except meringue was carried out using a chromometer at 0, 7, 14, and 28 d. The meringue was stored in a tightly closed container in the dark and analyzed after a period of 14 days. Longer times were not considered because of the limited shelf life.

Alcoholic beverage: 500 μL of the solution was added to 20 mL water and 6 g sucrose, heated, and stirred until the sugar was completely dissolved and then allowed to cool. Subsequently, 5 mL ethanol was added and stirred. The beverage was stored at room temperature in the dark, and samples were taken for color analysis as described above.

### 2.11. Instrumental Analysis

U*V*/*Vis* absorption spectra were recorded on a V-730 JASCO double-beam spectrophotometer (JASCO Deutschland GmbH, Pfungstadt, Germany) between 400 and 800 nm in a 1 cm path-length glass cuvette (Hellma GmbH & Co. KG, Müllheim, Germany). 

UHPLC analysis was conducted on a Prominence UFLC system (Shimadzu, Kyoto, Japan) equipped with two Nexera X2 LC-30AD high-pressure gradient pumps, a Prominence DGU-20A5R degasser, a Nexera SIL-30AC Prominence autosampler (15 °C, injection volume 5 μL), a CTO-20AC Prominence column oven at 40 °C, and an SPD-M20A Prominence diode array detector. An Acquity HSS-T3 RP18 column (150 mm × 2.1 mm; 1.8 μm particle size) was combined with a precolumn (Acquity UPLC HSS T3 VanGuard, 100 Å, 2.1 mm × 5 mm, 1.8 μm), both from Waters (Milford, MA, USA). The separation was performed with water (A) and acetonitrile (B) as eluents, both acidified with 0.1% (*v*/*v*) formic acid. A gradient elution program at a flow rate of 0.4 mL/min was used as follows (min/%B): 0/2; 3/14; 5/15; 6/30; 12/40; 12.1/100; 15/100; 15.1/2; 17/2.

UHPLC-MS analysis of the pigment was performed on an Acquity UPLC I-Class system (Waters, Milford, MA, USA) coupled with an LTQ-XL ion trap mass spectrometer (Thermo Scientific, Inc., Waltham, MA, USA), which was equipped with an electrospray interface operating in positive and negative ion mode. The apparatus consisted of a sample manager cooled at 10 °C, a binary pump, a column oven, and a diode array detector scanning from 200 to 600 nm. The column oven temperature was set at 40 °C. An Acquity HSS-T3 RP18 column (150 mm × 2.1 mm; 1.8 μm particle size) was combined with a precolumn (Acquity UPLC HSS T3 VanGuard, 100 Å, 2.1 mm × 5 mm, 1.8 μm), both from Waters (Milford, MA, USA). The separation was performed with water (A) and acetonitrile (B) as eluents, both acidified with 0.1% (*v*/*v*) formic acid. A gradient elution program at a flow rate of 0.4 mL/min was used as follows (min/%B): 0/2; 3/14; 5/15; 6/30; 12/40; 12.1/100; 15/100; 15.1/2; 17/2. The injection volume was 5 μL. 

Mass spectra were recorded in the range of *m*/*z* 300–800. For positive mode, the source voltage was kept at 4.5 kV at a current of 100 μA, and the tube lens was adjusted to 50 V. The capillary temperature was set at 350 °C with a spray voltage of 4.5 V. Nitrogen was used as the sheath, auxiliary, and sweep gas at a flow of 70, 10, and 1 arbitrary units, respectively. Xcalibur software (2.2SP1.48, Thermo Scientific, Inc., Waltham, MA, USA) was used for data analysis.

Color parameters according to CIELAB color metrics were determined using a CR-400/410 chromometer with illuminant D65 (Konica Minolta, Langenhagen, Germany). For this purpose, 15 mL of milk, oat drink, alcoholic beverages, and 15 g of yogurt were subjected to color measurements in a glass cuvette with a diameter of 60 mm. Color loss during storage was calculated as the overall color difference ΔE* and Δa obtained from the color parameters: L* indicates brightness, a* is the red (positive value)/green (negative value) coordinate, and b* is the yellow (positive value)/blue (negative value) coordinate.

## 3. Results and Discussion

As reported in our previous paper [16], the oxidation of CQA with NaIO_4_ in the presence of Trp for 72 h led to the formation of several products, three of them showing a red color. In an effort to accelerate pigment formation, the influence of various parameters on color development, as described in the following, was investigated using the areas of the three peaks detectable at 550 nm as a measured value.

### 3.1. Parameters Influencing the Development of the Red Pigment

#### 3.1.1. Temperature Effect

**Figure 2:**
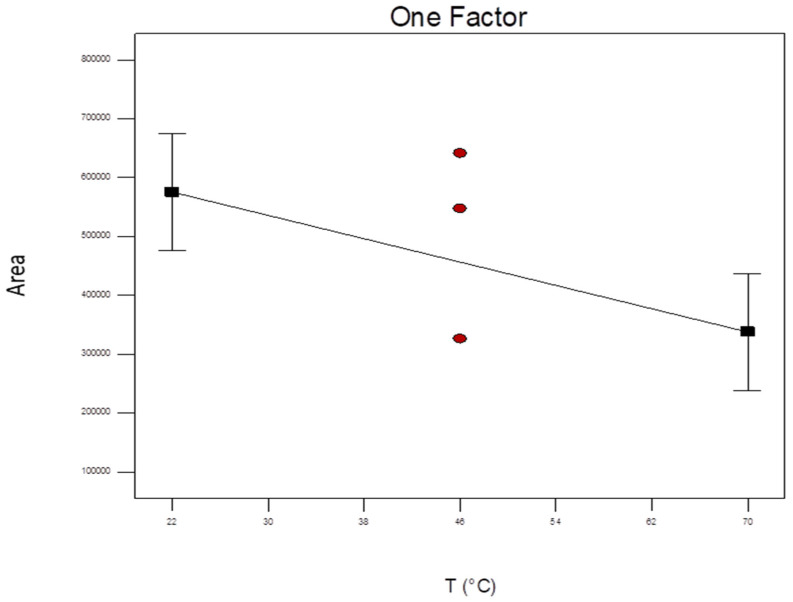
Variation of total peak area at 550 nm as a function of temperature after 24 h reaction time, with Trp and NaIO_4_ concentration on medium level (98 mM and 7.84 mM respectively). The red points represent the center points.

The one-factor plot of the factorial screening design shows that temperature has a significant linear and negative effect on the peak area of the red pigments (Figure 2). Concomitantly, the formation of a brown color hue at the cost of the red color was observed when the reaction was performed at higher temperatures. This may be explained by the preferred oxidation of chlorogenic acid and subsequent reactions of the quinone to high-molecular compounds before condensation with Trp to form the red pigment takes place [17]. From these findings, it can be concluded that in a range of 22–70 °C, there is a negative effect and, therefore, that the reaction should preferably be carried out at room temperature. While it might be of interest to test temperatures below this range, it needs to be considered that cooling would be required, which would entail additional energy costs and might compromise the feasibility of the process.

#### 3.1.2. Effect of Tryptophan Concentration

The effect of the Trp concentration is slightly positive (Figure S1). At the same time, we observed that Trp could not completely be dissolved when used in higher quantities. Therefore, it was decided to use Trp at a concentration of 56 mM.

#### 3.1.3. Effect of NaIO_4_ Concentration

**Figure 3:**
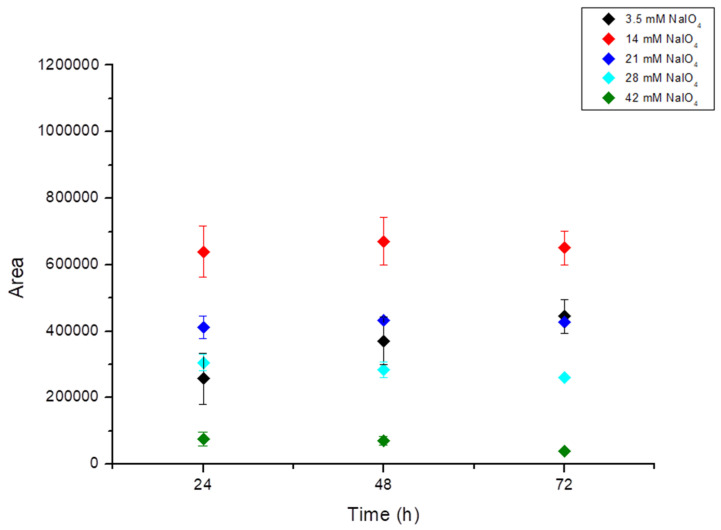
Variation of total peak area at 550 nm as a function of concentration of NaIO_4_ after 24, 48, and 72 h reaction time.

With increasing concentrations of NaIO_4_, a significant increase in the total area of the peaks absorbing at 550 nm was observed (Figure S2). Since no maximum could be identified, additional tests with higher NaIO_4_ concentrations (21, 28, and 42 mM) were conducted (Figure 3). However, as the increase did not lead to an improvement, the reaction seems to be less efficient. The highest yields of red pigments were observed when the concentration of NaIO_4_ was equal to that of CQA (14 mM, 1 M equivalents). The reaction with 3.5 mM (0.25 M equivalents) NaIO_4_ reacting at room temperature corresponds to that reported previously [16]. 

Statistical analysis of the central composite design resulted in a linear model (*p* = 0.0011 with a significant positive effect of NaIO_4_ (*p* ≤ 0.0003), satisfying regression coefficients (0.6–0.7), and no significance of the lack of fit. The optimization led to the following parameter settings: 14 mM CQA, 56 mM Trp, 14 mM NaIO_4_, 22 °C, and a reaction time of 24 h. 

#### 3.1.4. Effect of pH Value

**Figure 4:**
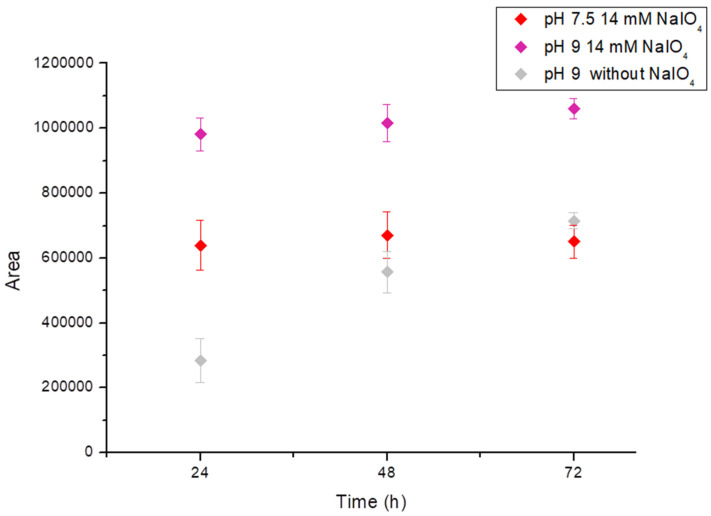
Total peaks area at 550 nm after 24, 48, and 72 h in the presence of 14 mM of NaIO_4_ (1 M equivalent) in Tris buffer 0.2 M at pH 7.5, at pH 9, and at pH 9 without the use of NaIO_4_.

The optimized reaction was subsequently conducted at different pH values (Figure 4). At pH 5, no red product was generated, whereas pH 9 was considered optimal for the development of the reaction. Although it was found that the reaction at pH 9 without NaIO_4_ also led to the formation of the red pigment with a chromatographic profile identical to that of the reaction with NaIO_4_, the reaction proceeded slower, and the yield after 72 h was comparable to that with 14 mM NaIO_4_ at pH 7.5 and even lower than that resulting from a combination of pH 9 and 14 mM NaIO_4_ (1 M equivalents) after 24 h.

In conclusion, the yield of red pigments was successfully increased by a combination of alkaline pH and the addition of NaIO_4_, while concomitantly, the reaction time was tremendously reduced from 72 h to 24 h. Compared to our previous study [16], this optimized method even achieved a slight increase in yield under these conditions.

### 3.2. Optimized Process for the Production of the Red Pigment

Based on the results of the above studies, the optimum reaction conditions were found to be room temperature (22 °C), Tris buffer 0.2 M (pH 9), and a ratio of 56 mM (4 M equivalents) of Trp and 14 mM (1 M equivalents) of 5-CQA in the presence of 14 mM (1 M equivalent) of NaIO_4_. To obtain a complete view of pigment formation, the reaction was carried out under these conditions, and peak areas were determined after 3, 6, 12, 24, 48, and 72 h. From Figure S3, it can be seen that after 24 h, significantly higher peak areas were obtained than after shorter reaction times, whereas a further increase to 48 h and 72 h did not lead to a considerable improvement. Therefore, a reaction time of 24 h can be considered optimal.

The scale-up of the reaction had no significant effect on its performance. After 24 h of reaction, a consumption of 89 ± 2% CQA and 48 ± 1% Trp was observed. The residual amount of CQA and Trp was determined by comparing the peak areas in the HPLC trace with those obtained by standard solutions of the compound at a known concentration. After the precipitation step with 6 M HCl, the pigment was centrifuged and lyophilized. HPLC analysis of the pigment (dissolved in an alkaline medium) showed the presence of 3% Trp, while no residual CQA was detected. 

For the first purification process, the pigment was dissolved in a minimum amount of alkaline solution and directly subjected to purification on a Sephadex G-10 column. The purification protocol conducted in triplicate proved to be reproducible, leading to yields of 46 ± 2% (*w*/*w*) with respect to the initial 5-CQA, and no residual Trp was detected by HPLC. 

### 3.3. Solubility of the CQA-Trp Pigment

The pigment obtained after the first purification step by size exclusion chromatography was not soluble in water but completely soluble in ethanol and 0.06 M NaOH. When the pigment dissolved in ethanol was further diluted with water, a suspension formed after a few minutes. It is assumed that after precipitation of the colorant with hydrochloric acid, the acidic environment still renders the compound insoluble when water is added. In contrast, when the colorant is first dissolved in an alkaline solution and then diluted with water, complete solubility is achieved. Prolonged exposure of the pigment to alkaline solutions decreased the peak intensity of all three isomers, favoring the formation of a single compound resulting from the release of the quinic acid moiety [16]. This conversion of the three red compounds to their hydrolyzed form was complete after 4 h.

### 3.4. Characterization of pH, Light, and Thermal Stabilities of the Hydrolyzed CQA-Trp Pigment

The hydrolyzed pigment stock solution was subsequently used to investigate its chemical and physical properties and thus assess its potential applications in the food industry. Because processing and storage may cause the isomerization and even the release of the chlorogenic acid moiety, which might, in turn, lead to a change in the physicochemical characteristics of the pigment, we preferred to use its hydrolyzed form in the application studies. 

**Figure 5:**
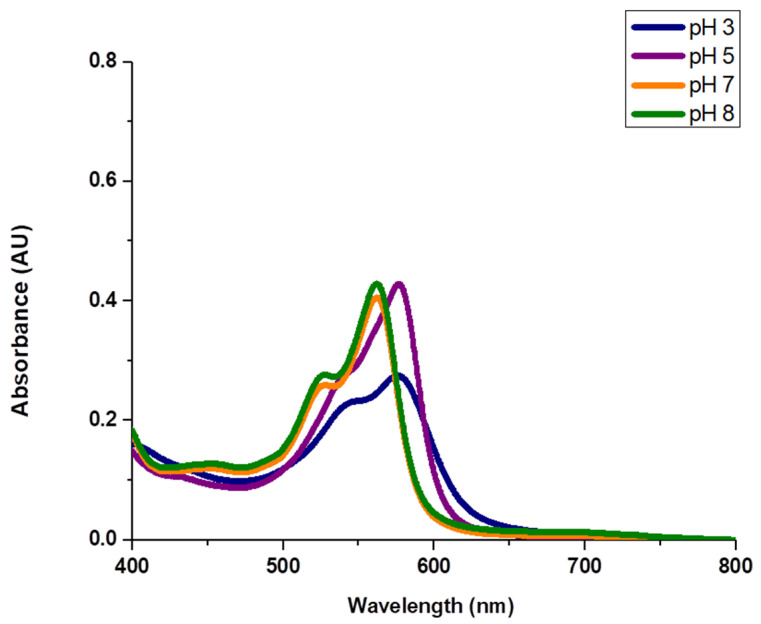
UV-Vis absorption spectra of solution 0.029 mg/mL of the red pigment in the hydrolyzed form in 0.1 M acetate buffer (pH 3 and 5) and in 0.1 M phosphate buffer (pH 7 and 8).

However, the authors concede that in some applications, the use of the pigment with the quinic acid moiety attached may be preferable because of its higher solubility. As shown in Figure 5, a pH dependence can be observed with a hypsochromic shift of 15 nm (from 577 to 562 nm) in the maximum absorption between pH 3 and pH 8. At pH 3, the color intensity decreased. In addition, when the pigment was exposed to acidic conditions (pH 3), it tended to form a precipitate over time, which confirms the previously reported low solubility of the colorant in an acidic solution. Remarkably, the precipitate could be redissolved by shaking and did not occur at higher pH values. Considerable differences in absorbance were not observed between pH values 7 and 8. The behavior of the pigment at higher pH values was not studied because alkaline conditions rarely occur in foods.

**Figure 6:**
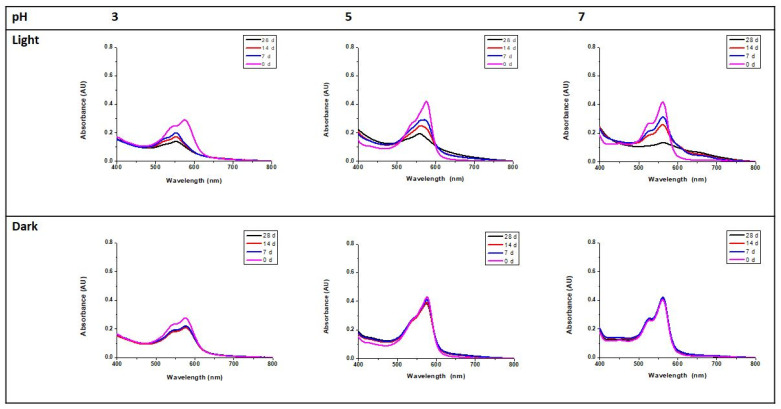
UV-Vis absorption spectra of solutions of the hydrolyzed pigment at different pH values and times (0/7/14/28 d) after exposure to light or dark.

The stability of the hydrolyzed CQA-Trp pigment during storage in the dark and when exposed to light was also tested. From Figure 6, it can be seen that the pigment was particularly stable for 28 days in the dark, whereas it was photosensitive as the period of light exposure increased. Data for pH 8 are not presented because no significant difference to pH 7 was found.

**Figure 7:**
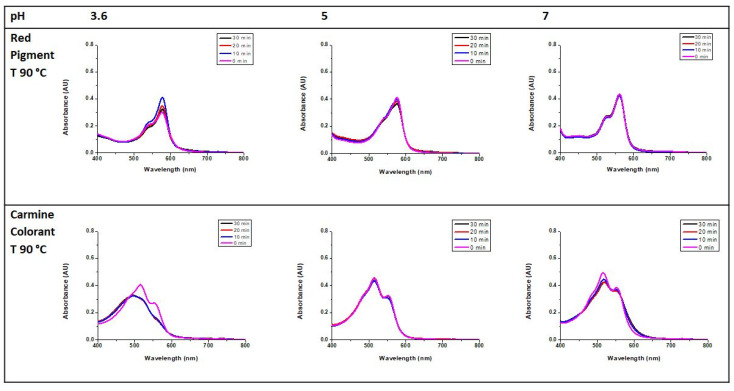
UV–Vis absorption spectra of solutions of the hydrolyzed pigment (0.029 mg/mL) and carmine (0.014 mg/mL) at different times in 0.1 M acetate buffer (pH 3.6 and 5) and 0.1 M phosphate buffer (pH 7).

The thermal stability of the hydrolyzed pigment was studied and compared to a commercial carmine preparation used in food applications. The pigment solutions were kept at 90 °C and analyzed by UV–Vis spectroscopy after 0, 10, 20, and 30 min (Figure 7). Their stability to thermal treatment was comparable. However, only half the concentration of the novel pigment was needed to obtain similar absorbance values. Remarkably, only at pH 3.6, the red pigment showed an increase in absorbance after 10 min. The reasons for this behavior are not entirely clear. However, an explanation might be an increase in the–initially limited–solubility under acidic conditions due to the effect of the temperature. Higher pH values did not lead to a remarkable difference in absorbance over time. The good stability of the pigment to heating under different pH conditions is an important advantage for a wide range of food applications, as this is one of the main limitations of natural pigments, which tend to degrade when subjected to heat [9].

### 3.5. Stability of Hydrolyzed Pigment against Reducing Compounds

No changes in absorbance were observed after mixing the pigment with ascorbic acid or cysteine at pH 3.6 for a period of 24 h. However, at pH 7 and in the case of ascorbic acid, a decrease in absorbance of 30% during 24 h was found, probably due to the acidity of ascorbic acid. With cysteine, no changes in absorbance occurred (Figure S4), which indicates that interactions with the thiol group that might affect color stability did not take place under the conditions investigated. This finding is extremely important because both ascorbic acid and cysteine are frequently found as natural components in foods and are also approved as additives. Furthermore, our results highlight the importance of the pH value of food matrices intended to be colored with the novel pigment.

### 3.6. Application as a Food Colorant

The potential of the pigment as a food colorant was explored using several food matrices. However, because of its insolubility in most of these matrices, it was necessary to dissolve the pigment in NaOH 0.06 M. This step also ensures total conversion to the hydrolyzed form. 

**Figure 8:**
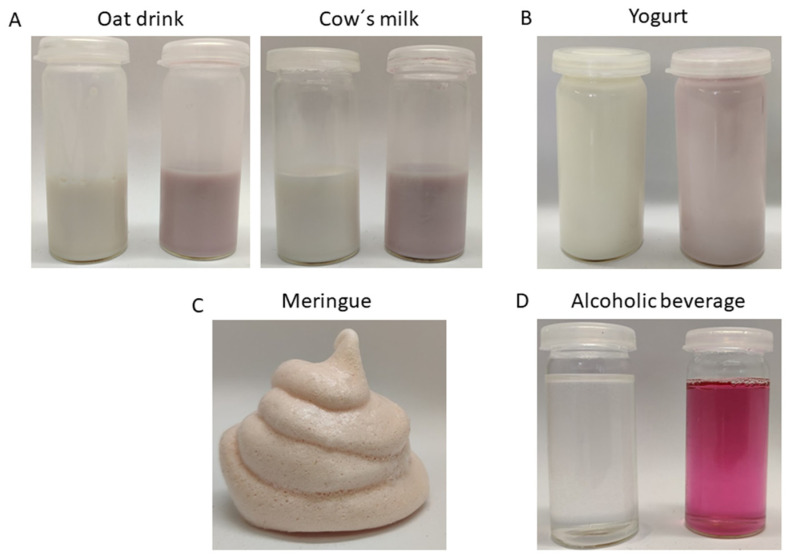
Food applications of the novel pigment. (**A**) Pure oat drink and oat drink with 0.01% *w*/*w* of colorant (left); Pure cow´s milk and cow´s milk with 0.01% *w*/*w* of dye. (**B**) Pure natural yogurt and natural yogurt with 0.005% *w*/*w* of colorant. (**C**) Meringue containing 0.007% *w*/*w* of colorant. (**D**) Alcoholic beverage with 20% ethanol containing 0.01% *w*/*w* of colorant.

Food application trials yielded more than satisfactory results with all model foods tested using the pigment in amounts less than 0.01% *w*/*w* (Figure 8).

Cow’s milk was selected as a model system because of its high commercial importance and tendency to be accepted with a reddish color, for example, when red berries such as strawberries are added. Plant-based drinks such as oat drinks are considered milk alternatives and have attracted increasing attention from consumers who wish to avoid animal-derived foods. Our studies revealed that the novel pigment produced excellent results for these specific applications, inducing a homogeneous color hue. Also, yogurt proved to be a suitable matrix because no precipitation occurred, and good pigment stability was observed under moderately acidic conditions. Finally, a potential use for an alcoholic beverage was proposed in light of the increased demand for drinks with vivid colors achieved by adding pigments, which appeal to a broad consumer base. Color stability in the samples was monitored for 28 days with CIELAB color space, with the exception of meringue, which was evaluated only for 14 days because of its limited storage stability. During this period, no significant changes in the a* values were observed (Table S4). 

## 4. Conclusions

The optimization of the pigment formation process reported in the present study has led to shorter production times and higher yields. This represents an important step for the commercial success of the novel pigment. Our investigations into the physicochemical properties indicate its potential suitability as a food colorant. However, as with all food additives, its use is subject to approval by the relevant authorities. Therefore, it is crucial to demonstrate its safety through comprehensive toxicological assessments and to address regulatory issues related to the inclusion of this compound in the list of approved food additives.

## Data Availability

The original contributions presented in the study are included in the article/Supplementary Materials, further inquiries can be directed to the corresponding author.

## Supplementary Materials

The following supporting information can be downloaded at: https://www.mdpi.com/article/10.3390/foods13050686/s1, Table S1: Experimental conditions for plan 1; Table S2: Experimental conditions for plan 2; Table S3: Experimental conditions for plan 3; Table S4: Values of color space for different model food applications. C represents a control without the addition of dye, while +P refers to the sample with the addition of colorant. ΔE* and Δ a were calculated using values from day 0 and day 28, with the exception of the meringue, which was calculated using values from day 0 and day 14; Figure S1: Variation of total peak area at 550 nm as a function of Trp concentration after 24 h reaction time; Figure S2: Variation of total peak area at 550 nm as a function of NaIO4 concentration after 24, 48 and 72 h reaction time; Figure S3: Area of the total peaks at 550 nm for the optimal reaction measured at 3, 6, 12, 24, 48, and 72 h; Figure S4: UV-Vis absorption spectra of red solution 0.029 mg/mL in 3.6 pH acetate buffer 0.1 M, pH 7 phosphate buffer 0.1 M or H_2_0 in presence of 1 mg/mL ascorbic acid, cysteine, or plain buffer solution.
