# Supplementary material for: Improved Generation, Physicochemical Characteristics, and Food Application Studies of a Red Colorant Obtained from Oxidative Coupling of Chlorogenic Acid and Tryptophan
Source: Foods. 2024 Feb 23;13(5):686. doi: 10.3390/foods13050686 (PMC10931162; doi:10.3390/foods13050686)
Supplement: Supplementary file 1 [file foods-13-00686-s001.zip › foods-2813641-supplementary.pdf]

Improved Generation, Physicochemical Characteristics, and Food Application Studies of a Red Colorant Obtained from Oxidative Coupling of Chlorogenic Acid and Tryptophan

Ardemia Santarcangelo, Nadine Schulze-Kaysers, and Andreas Schieber *

Institute of Nutritional and Food Sciences, Molecular Food Technology, Agricultural Faculty, University of Bonn, Bonn, Germany

* Corresponding author:

Prof. Dr. Andreas Schieber, Institute of Nutritional and Food Sciences, Molecular Food Technology, Agricultural Faculty, University of Bonn, Friedrich-Hirzebruch-Allee 7, D-53115 Bonn, Germany; email: schieber@uni-bonn.de; phone: +49 (0)228-73-4452

Email addresses:

Ardemia Santarcangelo: santarcangelo@uni-bonn.de

Nadine Schulze-Kaysers: nadine.schulze@uni-bonn.de

Andreas Schieber: schieber@uni-bonn.de

Supplementary Materials

Table S1: Experimental Conditions for plan 1. 3

Table S2: Experimental Conditions for plan 2. 4

Table S3: Experimental Conditions for plan 3. 5

Table S4: Values of CIELAB color space for different model food applications. C represents a control, without the addition of dye, while +P refers to the sample with addition of colorant. ***ΔE^**** and ***Δa*** were calculated using values from day 0 and day 28, with the exception of the meringue, which was calculated using values from day 0 and day 14. 7

Figure S1: Variation of total peak area at 550 nm as a function of Trp concentration after 24 h reaction time. 8

Figure S2: Variation of total peak area at 550 nm as a function of $NaIO_4$ concentration after 24,48 and 72 h reaction time. 10

Figure S3: Area of the total peaks at 550 nm for the optimal reaction measured at 3, 6, 12, 24, 48, and 72 h. 11

Figure S4: UV-Vis absorption spectra of red solution 0.029 mg/mL in 3.6 pH acetate buffer 0.1 M, pH 7 phosphate buffer 0.1 M or H_20 in presence of 1 mg/mL ascorbic acid, cysteine, or plain buffer solution. 12

Run-Order	CQA (mM)	Trp (mM)	$NaIO_4$ (mM)	T (°C)
1	14	98	7.84	46
2	14	56	1.68	70
3	14	56	14	70
4	14	98	7.84	46
5	14	56	1.68	22
6	14	140	1.68	22
7	14	140	1.68	70
8	14	56	14	22
9	14	140	14	70
10	14	98	7.84	46
11	14	140	14	22

Table S1: Experimental Conditions for plan 1.

Run-Order	CQA (mM)	Trp (mM)	$NaIO_4$ (mM)
1	14	56	3.5
2	14	140	3.5
3	14	56	3.5
4	14	56	14
5	14	98	8.75
6	14	98	8.75
7	14	140	14
8	14	98	8.75
9	14	56	14
10	14	140	3.5
11	14	140	14
12	14	98	8.75

Table S2: Experimental Conditions for plan 2.

Run-Order	CQA (mM)	Trp (mM)	$NaIO_4$ (mM)
1	14	140	8.75
2	14	98	14
3	14	140	14
4	14	98	8.75
5	14	56	3.5
6	14	140	3.5
7	14	98	8.75
8	14	56	14
9	14	98	8.75
10	14	56	8.75
11	14	98	8.75
12	14	98	8.75
13	14	98	3.5

Table S3: Experimental Conditions for plan 3.

Sample	***time***	***L****	***a****	***b****	***Δ E****	***Δa***
Milk	O d	46.30	-0.70	4.63		
	28 d	47.17	-0.49	4.72	0.90	0.21
MilK + P	0 d	35.03	8.37	-1.32		
	7 d	41.36	8.41	-2.02		
	14 d	41.22	8.20	-1.94		
	28 d	40.92	8.28	-1.96	0.85	-0.09
Oat Drink C	0 d	42.77	0.91	5.66		
	28 d	46.11	0.86	6.63	3.48	-0.05
Oat Drink+ P	0 d	35.68	9.40	-0.11		
	7 d	38.93	9.40	-0.34		
	14 d	38.65	9.09	-0.25		
	28 d	38.06	8.97	-0.19	2.42	-0.43
Yogurt C	0 d	67.06	-2.16	6.95		
	14 d	53.37	-1.55	5.48		
	28 d	58.87	-2.20	5.83	8.27	-0.04
Yogurt + P	0 d	42.39	6.08	-0.37		
	7 d	40.77	7.14	-0.67		
	14 d	40.10	6.81	-0.62		
	28 d	40.80	6.63	-0.60	1.70	0.55
20 % alcoholic beverage C	O d	50.25	-0.61	0.39		
	24 d	51.5	-0.30	0.55	1.30	0.3
20 % alcoholic beverage+ P	0 d	32.30	29.25	-2.24		
	7 d	35.64	25.26	-4.75		

	14 d	33.79	30.47	-3.27		
	28 d	33.54	29.61	-3.53	1.83	0.36
Meringue + P	O d	73.58	6.85	11.61		
	14 d	78.45	6.09	9.79	5.25	-0.76

Table S4: Values of CIELAB color space for different model food applications. C represents a control, without the addition of dye, while +P refers to the sample with addition of colorant. ***Δ E**** and ***Δ a*** were calculated using values from day 0 and day 28, with the exception of the meringue, which was calculated using values from day 0 and day 14.

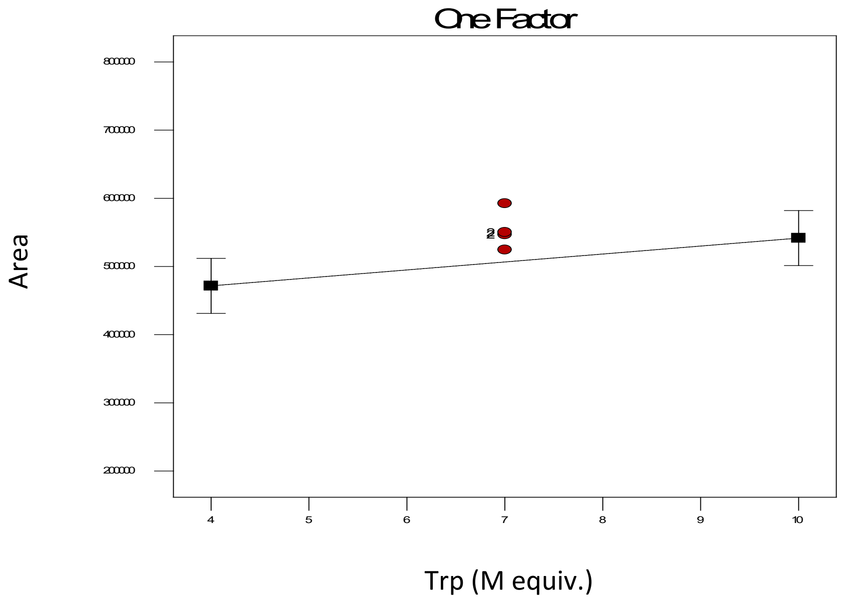

Figure S1: Variation of total peak area at 550 nm as a function of Trp concentration after 24 h reaction time with $NaIO_4$ concentration on medium level (8.75 mM). The red points represent the center points and 1M equivalent corresponds to 14 mM.

A) 24 h

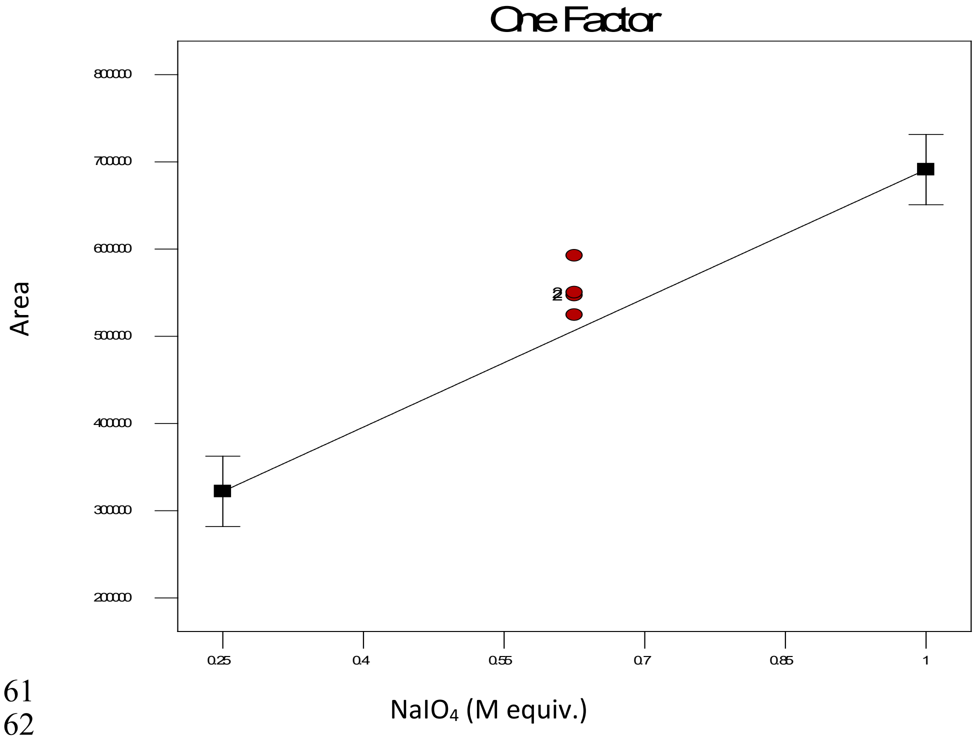

One Factor
Area
800000
700000
600000
500000
400000
300000
200000
2
0.25
0.4
0.55
0.7
0.85
1
NaIO4 (M equiv.)

B) 48 h

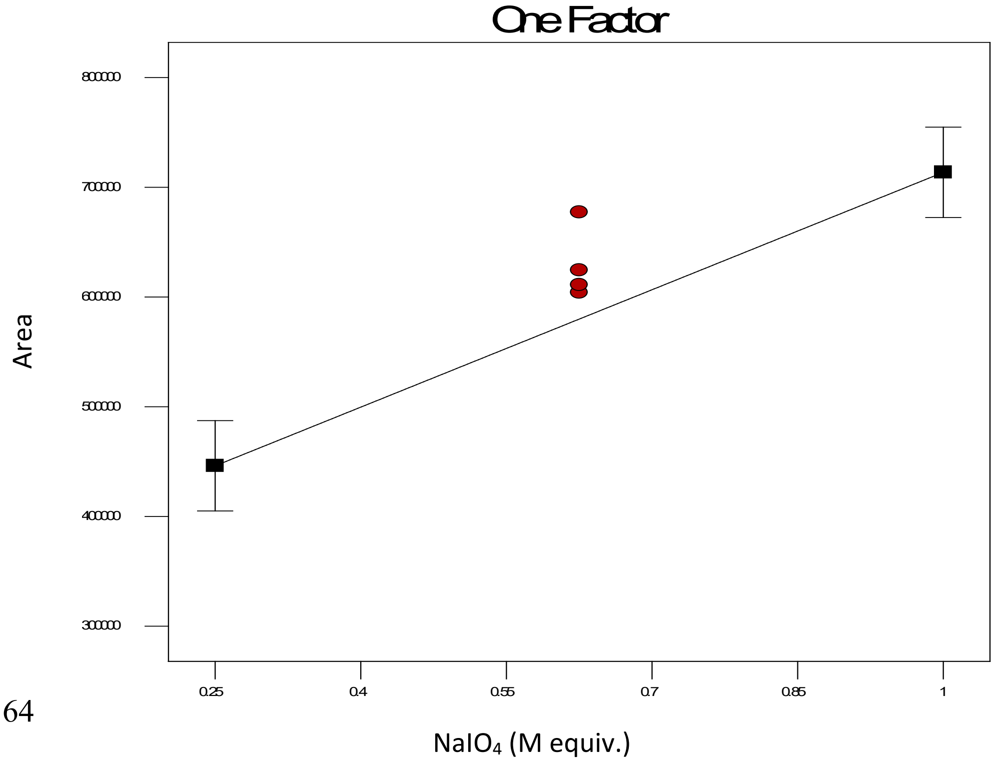

One Factor
Area
800000
700000
600000
500000
400000
300000
0.25
0.4
0.55
0.7
0.85
1
NaIO4 (M equiv.)

C) 72 h

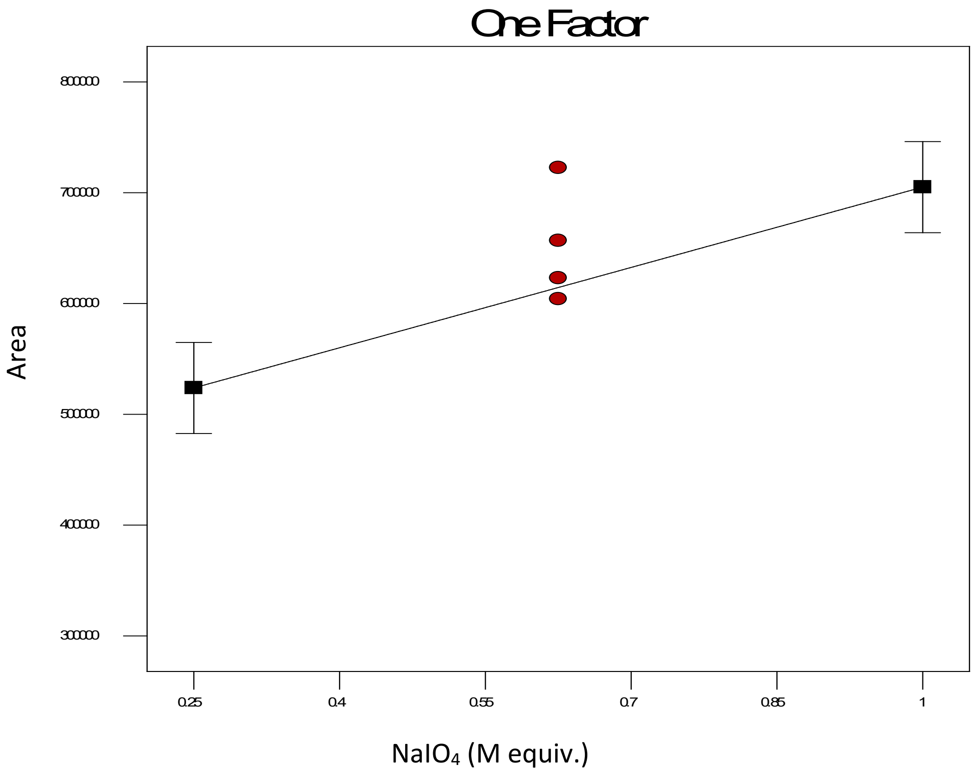

Figure S2: Variation of total peak area at 550 nm as a function of $NaIO_4$ concentration after 24, 48 and 72 h reaction time with Trp concentration on medium level (98 mM). The red points represent the center points and 1M equivalent corresponds to 14 mM.

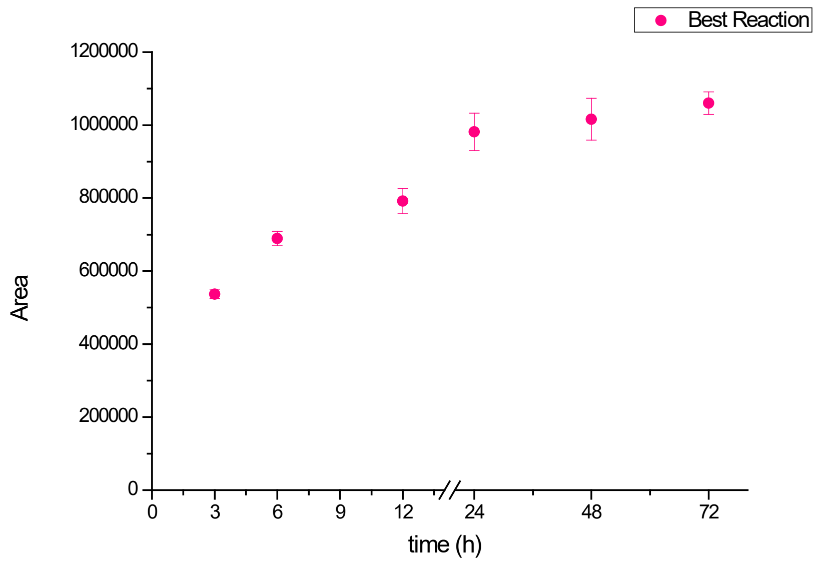

Figure S3: Area of the total peaks at 550 nm for the optimal reaction measured at 3, 6, 12, 24, 48, and 72 h.

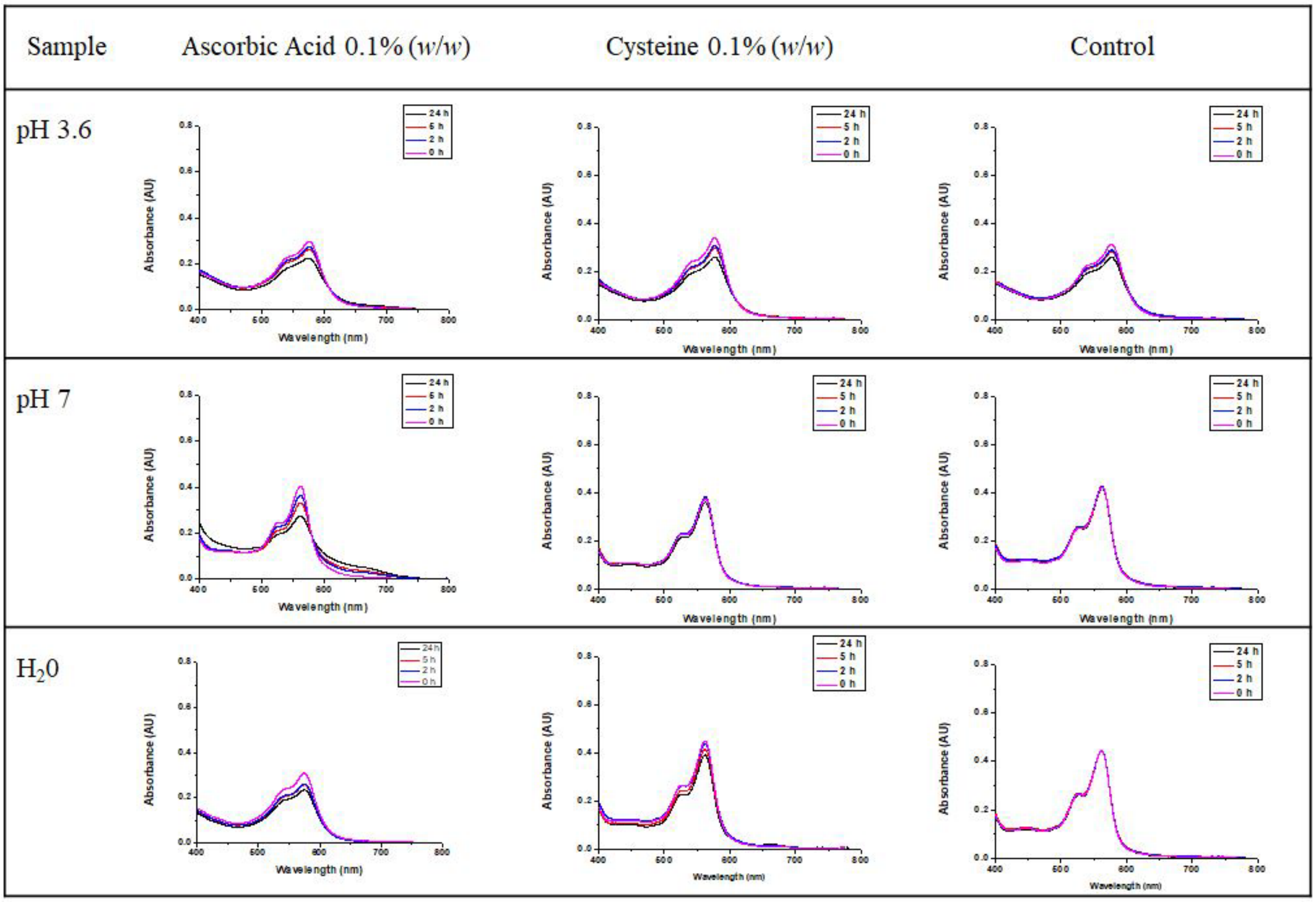

Figure S4: UV-Vis absorption spectra of red solution 0.029 mg/mL in 3.6 pH acetate buffer 0.1 M, pH 7 phosphate buffer 0.1 M or H_20 in presence of 1 mg/mL ascorbic acid, cysteine, or plain buffer solution.
